# Early Warning Information for Severe and Critical Patients With COVID-19 Based on Quantitative CT Analysis of Lung Segments

**DOI:** 10.3389/fpubh.2021.596938

**Published:** 2021-05-13

**Authors:** Xu Yuyun, Yu Lexi, Wang Haochu, Shu Zhenyu, Gong Xiangyang

**Affiliations:** ^1^Department of Radiology, Zhejiang Provincial People's Hospital, Affiliated People's Hospital, Hangzhou Medical College, Hangzhou, China; ^2^Wuhan Wuchang Hospital, Wuchang Hospital Affiliated to Wuhan University of Science and Technology, Wuhan, China

**Keywords:** coronavirus, COVID-19, lung, prognosis, severe acute respiratory syndrome

## Abstract

**Background:** The coronavirus disease 2019 (COVID-19) outbreak is spreading rapidly around the world.

**Purpose:** We aimed to explore early warning information for patients with severe/critical COVID-19 based on quantitative analysis of chest CT images at the lung segment level.

**Materials and Methods:** A dataset of 81 patients with coronavirus disease 2019 (COVID-19) treated at Wuhan Wuchang hospital in Wuhan city from 21 January 2020 to 14 February 2020 was retrospectively analyzed, including ordinary and severe/critical cases. The time course of all subjects was divided into four stages. The differences in each lobe and lung segment between the two groups at each stage were quantitatively analyzed using the percentage of lung involvement (PLI) in order to investigate the most important segment of lung involvement in the severe/critical group and its corresponding time point.

**Results:** Lung involvement in the ordinary and severe/critical groups reached a peak on the 18th and 14th day, respectively. In the first stage, PLIs in the right middle lobe and the left superior lobe between the two groups were significantly different. In the second stage and the fourth stage, there were statistically significant differences between the two groups in the whole lung, right superior lobe, right inferior lobe and left superior lobe. The rapid progress of the lateral segment of the right middle lobe on the second day and the anterior segment of the right upper lobe on the 13th day may be a warning sign for severe/critical patients. Age was the most important demographic characteristic of the severe/critical group.

**Conclusion:** Quantitative assessment based on the lung segments of chest CT images provides early warning information for potentially severe/critical patients.

## Introduction

The coronavirus disease 2019 (COVID-19) outbreak was declared a global health emergency by the WHO on 30 January 2020. As the new coronavirus is far more transmissible than SARS and MERS, it has led to outbreaks in many countries and regions around the world. According to the latest report of the World Health Organization, more than twelve million people have been infected, and about 2.8 million patients have died from COVID-19 pneumonia^1^, with a fatality rate of around 1–3% (1). Although the mortality rate of COVID-19 is much lower than the 9.6% of SARS and 34.4% of MERS, its strong pathogenicity is notable.

According to the Diagnosis and Treatment of Novel Coronavirus Pneumonia (trial version seven) of China, COVID-19 patients can be classified into mild, ordinary, severe, and critical cases (2). Among them, severe and critical patients are at a higher risk of acute respiratory distress syndrome, especially those with a severe onset and fast progression. If not treated in time, the ordinary cases may develop into severe/critical cases or even progress to death (3), so early warning of potentially severe/critical patients may further improve their prognosis. CT examination, as an essential method for the detection and assessment of COVID-19 pneumonia, provides essential information for clinical decision-making (4). Generally, CT imaging manifestations correspond to the severity of COVID-19 pneumonia (5); however, due to the potential radiation damage, CT scans should not be performed too often. Therefore, it is essential to optimize the timing of CT scans for early warning of disease deterioration with less radiation exposure, to help clinicians manage patients quickly and accurately (6).

Recent works have reported the characteristic imaging features of COVID-19 pneumonia, such as ground-glass opacities in the early stage, consolidation, and “crazy-paving” patterns in the peak stage, subpleural distribution, etc. (7, 8). However, due to the lack of accurate quantification tools, these radiological reports mainly used qualitative descriptions for lung involvement. Although there have been studies that used semi-quantitative methods such as a CT score to show the lung changes during the time course of COVID-19 pneumonia, it is very subjective, time-consuming, and it has low inter-rater reliability. In particular, Pan Fen described the imaging symptoms of the four stages of the lung in detail, but a method based on artificial visual assessment may not be able to mine additional potential information about the disease, and the assessment of the disease from the whole lung may not reveal the detailed progress of the disease (9). Therefore, it is necessary to introduce a fully automatic and quantitative method to provide clinicians with a method for accurate evaluation to guide efficient intervention and treatment. In addition, a quantification based on the segmental and lobar lung may help in understanding the precise development of COVID-19 pneumonia.

With growing global concerns about the COVID-19 outbreak, an accurate understanding of the evolution of chest imaging findings and the correlated timepoints are essential for effective patient management and treatment. Therefore, the purpose of our study is to explore the specific lung segments and possible transition times of progressive lesions and to identify potential severe/critical pneumonia patients early to further reduce the related mortality. We compared the differences in segmental distribution and longitudinal changes between severe/critical and ordinary COVID-19 patients based on a quantitative analysis at the lung segment level.

## Materials and Methods

### Study Population

This study was approved by the institutional review board and the local ethics committee of Zhejiang Provincial People's Hospital and Wuhan Wuchang hospital. All investigations were conducted under the Declaration of Helsinki. Written informed consent was waived for this retrospective study, which involved no potential risk to the patients. The dataset in this study was collected from Wuhan Wuchang hospital for COVID-19 patients in Wuhan city. Patients with COVID-19 pneumonia were diagnosed by real-time reverse-transcriptase polymerase chain reaction of throat swabs or lower respiratory tract samples from 24 January 2020 to 14 February 2020. Their information was retrospectively analyzed, including demographic data, initial clinical symptoms, history of underlying diseases, CT imaging data, and laboratory test data. According to the Diagnosis and Treatment of Novel Coronavirus Pneumonia (seventh trial version) of China (2), all subjects were divided into ordinary patients (*n* = 45) and severe/critical patients (*n* = 36), including six critical cases. The starting point of the longitudinal study was defined as the time point of symptom onset, and the endpoint was set as discharge, transfer, or death. In addition, there were no patients with negative imaging findings and no pediatric patients in the dataset. Specific grouping details can be found in the Supplementary Material. The coinfection of bacterial or viral pneumonia during hospitalization were recorded including the Nine joint tests for respiratory pathogens (antibodies): influenza B virus, Q fever rickettsiae, influenza A virus, adenovirus, mycoplasma pneumoniae, Legionella pneumoniae, respiratory syncytial virus, Chlamydia pneumoniae, Parainfluenza virus.

### CT Scanning Parameters

All patients underwent multiple non-contrast chest CT scans using a single inspiratory phase on a multi-detector CT scanner (Philips Medical Systems, Cleveland). Patients were instructed about breath-holding. CT images were then acquired during a single breath-hold. The tube voltage was set at 120 kVp during the CT acquisition. The CT images were reconstructed on the raw data with a matrix size of 512 × 512 as axial images (thickness of 2 mm and increment of 2 mm). The mean volume CT dose index range was 5.2–12.6 mGy. The scan range was from the apex of the lungs to the level of the costal angle of the base of the lungs.

### CT Image Assessment

Two chest radiologists (Y.L. and H.W., with ~20 years of experience in thoracic imaging), reviewed all of the CT scans independently and reached decisions by consensus. They were aware that the patients had COVID-19 pneumonia but were blinded to information concerning the patients' clinical conditions, such as laboratory results and the severity of symptoms and signs. Each of the five lung lobes was assessed for the degree of involvement and scored as 0 for 0%, 1 for 1–25%, 2 for 26–50%, 3 for 51–75%, or 4 for 76–100%. A total lung involvement severity score was reached by summing the five lobe scores (range of possible scores, 0–20) (8). All CT data were imported into AI software (care.ai® Intelligent Evaluation System, Version 6) for automatic lung involvement detection and quantitative analysis. The parameter of the percentage of lung involvement (PLI) was selected, which is defined as the ratio of pneumonia volume to the whole/segmental lung, as it is close to the CT score due to their similarity in assessing lung involvement by ratio to analyse the lung condition of five lung lobes and the corresponding 18 subordinate lung segments. Details about the 18 lung segments and the pneumonia assessment software can be found in the Supplementary Material. Finally, a correlation analysis was performed between the CT scores and the parameter of PLI for their reliability.

### Longitudinal Assessment of Lung Involvement

We divided the time course into four stages according to the quartiles of the CT scans of the two groups of patients. All CT duration times were sorted orderly first, and then we took the quartiles (25%, median, 75%, 100%) as the corresponding stage. The lung involvement of the lung lobes and segments of the ordinary and severe/critical groups at each stage were compared to identify the specific segment location of significant differences. Then, the specific segment that contributed most to the corresponding lung lobe involvement of each stage was selected using the ROC curve. Finally, a longitudinal analysis was performed on the PLIs of the specific segments. Thus, the specific lung involvement segment and its corresponding progression time point for severe/critical cases were identified.

### Statistical Analysis

The Statistical Package for Social Sciences (SPSS) version 25.0 (SPSS, Inc., Chicago, IL, USA) software package was used for statistical analysis. The Kolmogorov–Smirnov test was used for normality testing of the measurement data. The normally distributed data were evaluated using the independent sample *t*-test, whereas the non-normally distributed data were evaluated using the Mann–Whitney U test. The differences between categorical variables were tested by the chi-square test. Continuous variables from the clinical information were described using mean, median, and interquartile range (IR), and continuous variables from the CT image dataset were described using mean ± standard deviation. The results with a two-tailed *P* < 0.05 were considered significant.

## Results

### Demographic Data

Of the 81 patients with COVID, 55 (58%) were male, and their average age was 55 (IR, 46, 67; range, 21–93). The median time from initial symptoms to admission was 6 days (IR, 4, 8; range, 1–20 days), the average time from initial symptoms to the first CT scan was 9 days (IR, 5, 13; range, 0–25 days), and the average hospitalization time was 20 days (IR, 16, 25; range, 8–33). The main symptom among the initial symptoms was a fever in 80 (96.4%) cases, followed by a cough in 71 (87.7%) cases, then fatigue in 49 (60.5%) cases. Among all the patients, 12 cases were confirmed with other infections, including one Legionella pneumophila pneumonia, one Klebsiella pneumonia, one multiple bacterial pneumonia, one mycoplasma pneumonia, one mycoplasma pneumonia with adenovirus infection, two adenovirus infections, two parainfluenza virus infections, one respiratory syncytial virus infection, and two influenza B virus infections. There was no significant difference in the number of coinfection cases between ordinary patients and severe/critical patients.

The most common underlying disease was hypertension, with a total of 21 (25.9%) cases. The number with wet and dry rales on clinical physical examinations of the lung were 57 (70.4%). The C-reactive protein on laboratory tests was increased with an average of 34.95 (IR, 11.84–48.22). The white blood cell count was slightly lower, with a median of 3.51 (IR, 2.96, 4.88), and the percentages of neutrophils and lymphocytes were low, with median values of 77.7 (IR, 60.4–84.1) and 15.14 (IR, 10.05–22.05), respectively. There was a statistically significant difference in age between ordinary patients and severe/critical patients [50 (IR, 40–58; range 28–72 vs. 63 (IR, 52–71; range, 34–93 years old), *P* < 0.05], while the other clinical data were not significantly different (*P* > 0.05). Six of the patients died eventually, and the other 75 patients were cured and discharged. See Table 1 for details.

**Table 1:** Baseline clinical information of the study population.

**Characteristic**	**Study Population** **(*n* = 81)**	**Ordinary group** **(*n* = 45)**	**Severe/** **critical group** **(*n* = 36)**	***P*-value**
**Demographics**				
Age (y)	55 (46, 67)	50 (40, 58)	63 (52, 71)	<0.001
Male sex, *n* (%)	47 (58)	24 (53.3)	23 (63.9)	0.339
Heart rate, (n/minute)	80 (78, 96)	82 (78, 105)	80 (76, 91)	0.285
Respiratory frequency, (n/minute)	20 (20, 23)	20 (20, 23)	20 (20, 23)	0.282
Time between initial symptoms and hospitalization, (days)	6 (4, 8)	6 (4, 9)	6 (3, 8)	0.619
Time from initial symptom to first CT scan, (days)	9 (5, 31)	10 (5,14)	7 (4, 11)	0.36
Days of hospitalization, (days)	19 (16, 24)	20 (16, 24)	18 (15, 24)	0.437
**Initial clinical symptoms**, ***n*** **(%)**				
Fever	80 (96.4)	44 (97.7)	36 (100)	0.368
Cough	71 (87.7)	40 (88.9)	31 (86.1)	0.706
Expectoration	27 (33.3)	12 (26.7)	15 (41.7)	0.155
Chest pain and tightness	18 (22.2)	8 (17.8)	10 (27.8)	0.282
Dizziness and headache	16 (19.8)	8 (17.8)	8 (22.2)	0.618
Nausea and vomiting	7 (8.6)	5 (11.1)	2 (5.6)	0.377
Myalgia	12 (14.8)	8 (17.8)	4 (11.1)	0.401
Weakness	49 (60.5)	31 (68.9)	18 (50)	0.084
Abdominal pain and diarrhea	5 (6.2)	3 (6.7)	2 (5.6)	0.836
Rhonchi (*n*/%)	57 (70.4)	29 (64.4)	28 (77.8)	0.192
Coinfection	12 (14.8)	5 (11.1)	7 (19.4)	0.294
**History of underlying diseases**, ***n*** **(%)**				
Hypertension	21 (25.9)	14 (31.1)	7 (19.4)	0.234
Diabetes	11 (13.6)	5 (11.1)	6 (16.7)	0.468
Liver injury	17 (21)	9 (20)	8 (22.2)	0.807
Nephropathy	5 (6.2)	1 (2.2)	4 (11.1)	0.099
**Laboratory test**				
C-reactive protein (mg/L)	34.95 (11.84, 48.22)	30.88 (10.7, 51.6)	26.69 (12.94, 45.6)	0.775
Leukocyte ratio (%)	3.51 (2.96, 4.88)	3.68 (2.86, 5.03)	3.49 (2.99, 4.48)	0.864
Neutrophil ratio (%)	77.7 (60.4, 84.1)	77.7 (62.2, 84.8)	77.25 (58.43, 81.98)	0.537
Lymphocyte ratio (%)	15.14 (10.05, 22.05)	15.5 (9.8, 22.1)	14.1 (10.43, 24.48)	0.977

### The Stages and Dynamic Changes of Lung Involvement

**Figure 1:**
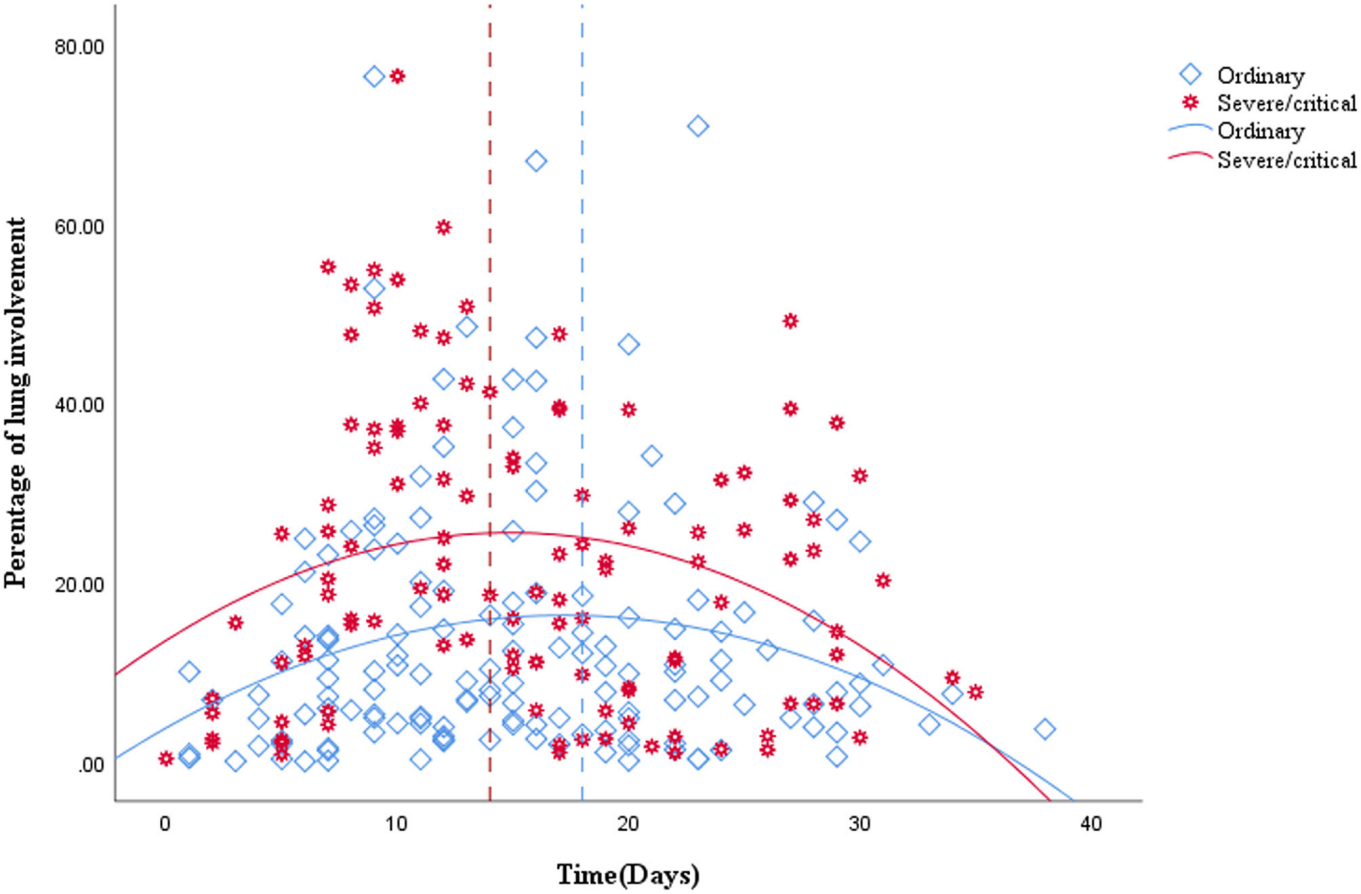
Quadratic scatter plot of percentages of lung involvement changes over time in the ordinary group and the severe/critical group. The dotted line represents the peak time of lung involvement in each group. The fitted line represents the change of lung involvement over time. The fit line formula for the ordinary group is Y = 3.74 + 1.46*X – 0.04*X2 (R2 = 0.049, *P* = 0.026), while the fit line formula for the severe/critical group is Y = 13.5 + 1.62*X – 0.05*X2 (R2 = 0.068, *P* = 0.019), in which x = days from the onset of the initial symptoms, y =total percentage of lung involvement of bilateral lungs. According to the fit line plot, the time point of maximum lung involvement for the ordinary group and severe/critical group was about the 18th day and 14th day, respectively.

A total of 265 CT scans were performed in 81 patients during their hospitalization. According to the quartile of all of the ordinary patients' 149 CT scans, the stages for the ordinary cases were as follows: the first stage on days 0–9, the second stage on days 10–14, the third stage on days 15–20, and the fourth stage from day 21 to the endpoint. By the quartile of all of the severe/critical patients' 116 CT scans, the first stage for the severe/critical group was on days 0–9, the second stage on days 10–15, the third stage on days 16–22, and the fourth stage from day 23 to the endpoint. The fitted curve of the PLI shows that pulmonary lesions reached their maximum severity on the 18th day for the ordinary group, while it was the 14th day for the severe/critical group. See Figure 1 for details.

### CT Quantitative Evaluation of Lung Lobes

**Figure 2:**
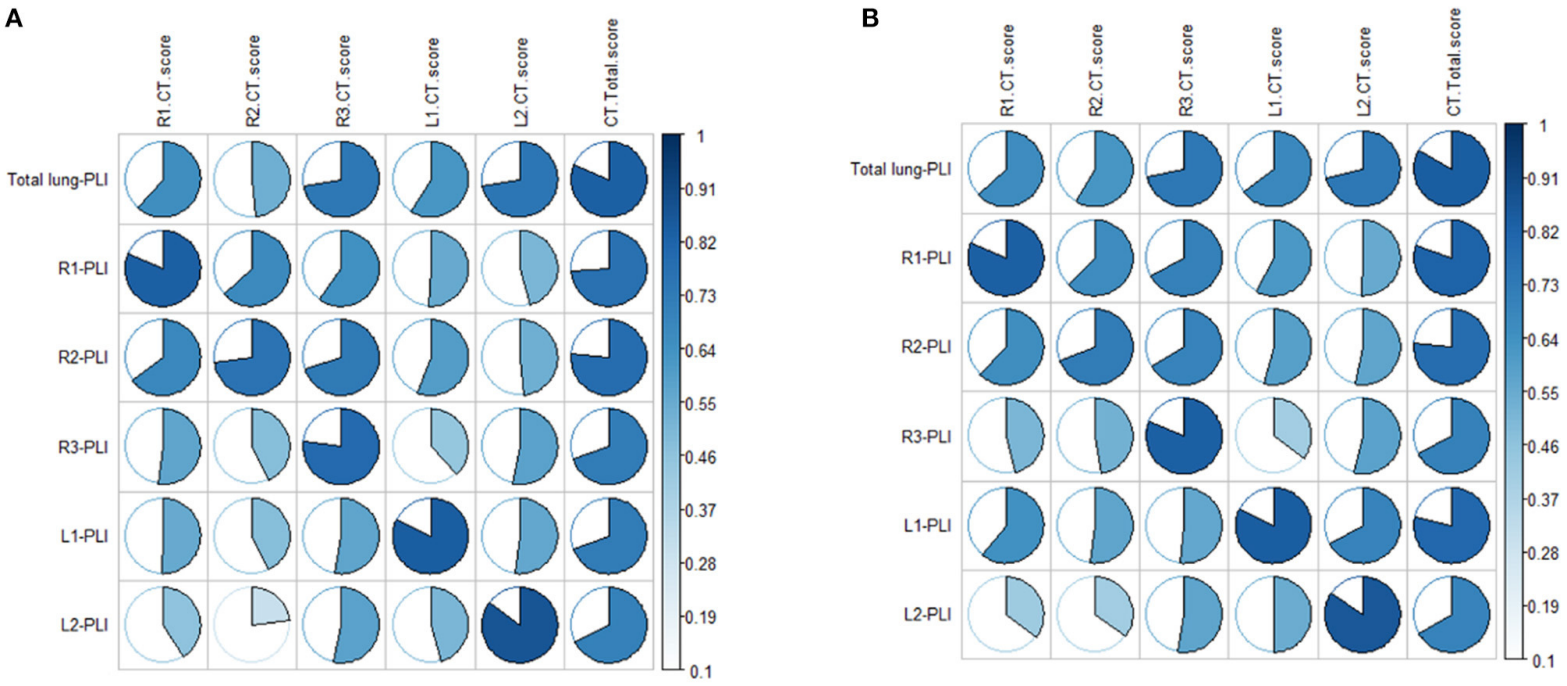
**(A,B)** Show the correlations between CT score and automatic quantitative parameter PLI in the ordinary group and the severe/critical group, respectively. The more complete the circle, the higher the correlation between the CT score and PLI. PLI, percentage of lung involvement; R1, right upper lobe; R2, right middle lobe; R3, right lower lobe; L1, left upper lobe; L2, left lower lobe.

**Figure 3:**
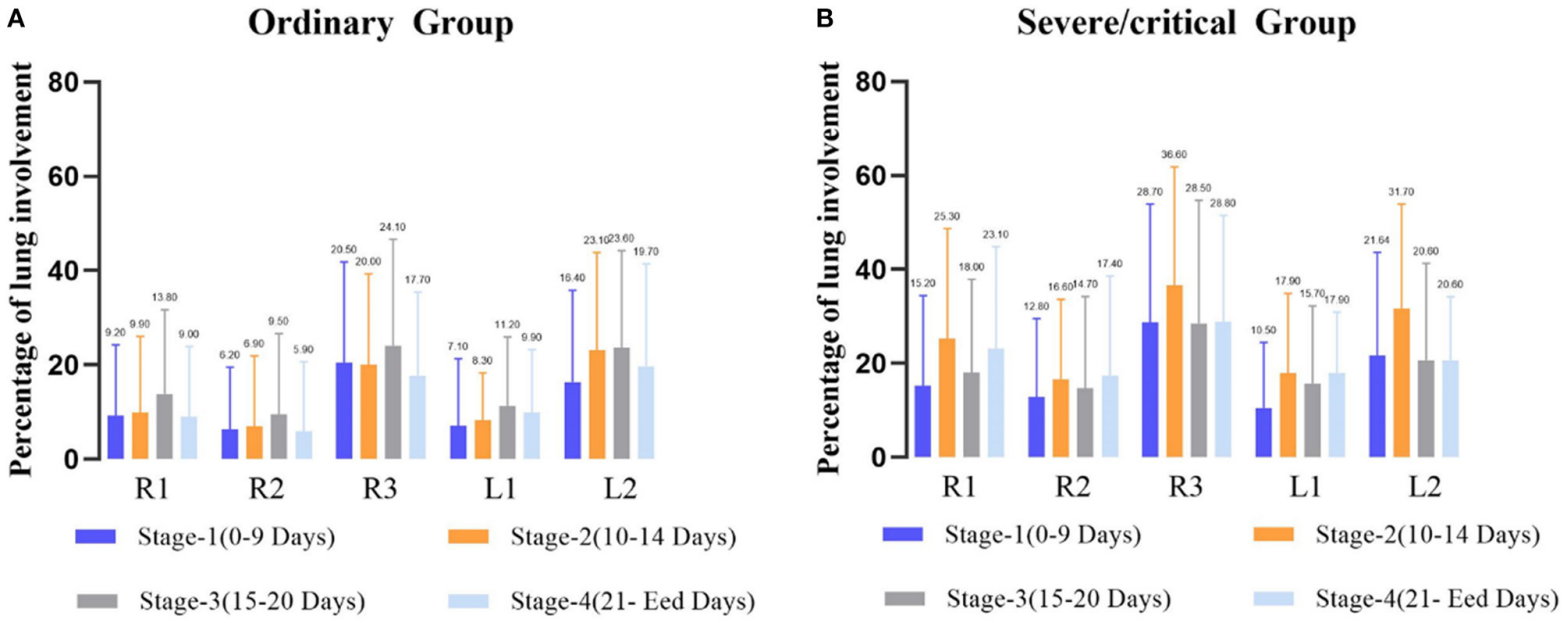
Average percentage of lung involvement of the five lobes at different time stages in the ordinary group **(A)** and the severe/critical group **(B)**. R1, right upper lobe; R2, right middle lobe; R3: right lower lobe; L1: left upper lobe; L2: left lower lobe.

Correlation analysis between the CT score and the PLI value showed a high correlation in both the ordinary group and the severe/critical group, with the correlation coefficient of each lung lobe >0.8, as shown in Figure 2. In the first stage, there was a statistically significant difference in PLI between the right middle lobe and the left superior lobe of the two groups (6.2 ± 13.3 vs. 12.8 ± 16.7, 7.1 ± 14.2 vs. 10.5 ± 13.9, *P* < 0.05). In the second stage, there was a statistically significant difference in the whole lung, right superior lobe, right inferior lobe and left superior lobe (13.3 ± 12.3 vs. 25.4 ± 19.8, 9.9 ± 16.1 vs. 25.3 ± 23.4, 20 ± 19.3 vs. 36.6 ± 25.2, 8.3 ± 10 vs. 17.9 ± 17.9, *P* < 0.05). The lung lobes with significant differences in the fourth stage were the same as those in the second stage (12.2 ± 13.4 vs. 21 ± 15.2, 9 ± 14.9 vs. 23.1 ± 21.7, 17.7 ± 17.7 vs. 28.8 ± 22.7, 9.9 ± 13.3 vs. 17.9 ± 13, *P* < 0.05). There was no significant difference in PLI in all lobes of the lung in the third stage (*P* > 0.05), as shown in Table 2 and Figure 3.

**Table 2:** Percentages of lung involvement in different stages of the ordinary and severe/critical groups.

	**Stage-1**		**Stage-2**		**Stage-3**		**Stage-4**	
	**Ordinary group** **(*n* = 42)**	**Severe/critical** **group (*n* = 32)**	**Ordinary group** **(*n* = 32)**	**Severe/critical** **group (*n* = 27)**	**Ordinary group** **(*n* = 39)**	**Severe/critical** **group (*n* = 31)**	**Ordinary group** **(*n* = 36)**	**Severe/critical group (*n* = 26)**
PLI of bilateral lungs (%)	11.9 ± 14.7	17.3 ± 17.2	13.3 ± 12.3	25.4 ± 19.8	16.4 ± 16.1	19.4 ± 17.4	12.2 ± 13.4	21 ± 15.2
*P*-value	0.131		0.011^*^		0.582		0.015^*^	
PLI of R1 (%)	9.2 ± 15	15.2 ± 19.2	9.9 ± 16.1	25.3 ± 23.4	13.8 ± 17.9	18 ± 19.9	9 ± 14.9	23.1 ± 21.7
*P*-value	0.058		0.003^*^		0.566		0.005^*^	
PLI of R2 (%)	6.2 ± 13.3	12.8 ± 16.7	6.9 ± 15	16.6 ± 17	9.5 ± 17.1	14.7 ± 19.5	5.9 ± 14.7	17.4 ± 21.2
*P*-value	0.003^*^		0.011		0.442		0.051	
PLI of R3 (%)	20.5 ± 21.3	28.7 ± 25.2	20 ± 19.3	36.6 ± 25.2	24.1 ± 22.5	28.5 ± 26.2	17.7 ± 17.7	28.8 ± 22.7
*P*-value	0.114		0.008^*^		0.425		0.046^*^	
PLI of L1 (%)	7.1 ± 14.2	10.5 ± 13.9	8.3 ± 10	17.9 ± 17.9	11.2 ± 14.7	15.7 ± 16.5	9.9 ± 13.3	17.9 ± 13
*P*-value	0.047^*^		0.013^*^		0.135		0.005^*^	
PLI of L2 (%)	16.4 ± 19.4	21.64 ± 22	23.1 ± 20.7	31.7 ± 22.2	23.6 ± 21.1	20.6 ± 20.7	19.7 ± 21.7	20.6 ± 13.5
*P*-value	0.27		0.153		0.591		0.185	

*PLI, percentage of lung involvement; R1, right upper lobe; R2, right middle lobe; R3, right lower lobe; L1, left upper lobe; L2, left lower lobe.*

* *p < 0.05*.

### CT Quantitative Evaluation of Lung Segments

**Figure 4:**
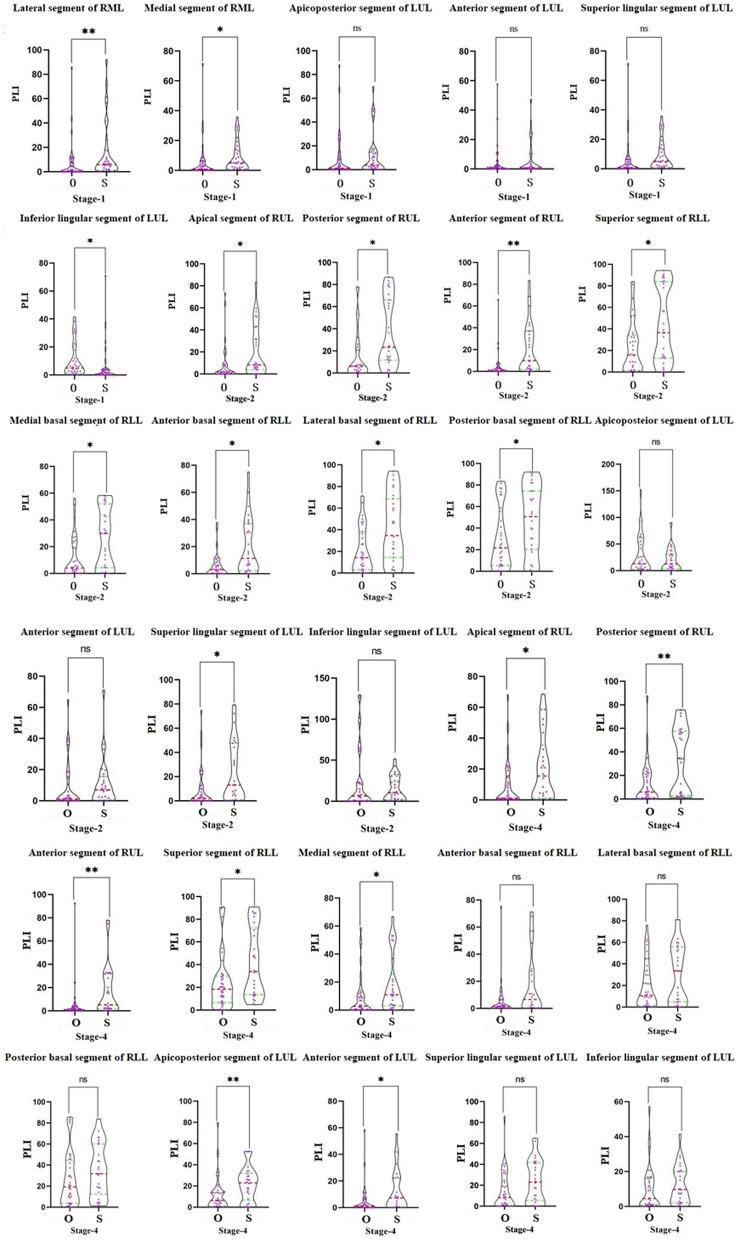
The comparison of the percentage of lung involvement of 18 lung segments in five lung lobes between the ordinary group and the severe/critical group. The red dotted line represents the median, and the green dotted line represents the quartile. O means ordinary group and S means severe/critical group. PLI, percentage of lung involvement; RML, right middle lobe; LUL, left upper lobe; RUL, right upper lobe; RLL, right lower lobe. *P < 0.05, **P < 0.01.

In the first stage, there was a statistically significant difference in the PLIs in the lateral and medial segments of the right middle lobe and the inferior lingular segment in the left upper lobe (*P* < 0.05) in the two groups. In the second stage, the PLIs of the ordinary and severe/critical groups were significantly different in the apical, posterior, and anterior segments of the superior lobe of the right lung, all five segments in the right inferior lung, and the superior lingular segment of the left superior lung. In the fourth stage, PLIs between the two groups in the apical, anterior, and posterior segment of the right superior lobe and superior and the medial basal segment of the right inferior lobe were significantly different. See details in Figure 4.

The ROC curve shows the greatest diagnostic power in the lateral segment of R2 in the first stage, anterior segment of R1 in the second stage, and posterior segment of the right upper lobe in the fourth stage with AUC, sensitivity, and specificity respectively of 0.721, 0.755, 0.734; 0.937, 0.556, 0.577; and 0.5, 0.906, 0.889. See Table 3 for details.

**Table 3:** Diagnostic efficacy of percentage of lung involvement in the lung segments in different stages.

	**Name of lung segment**	**AUC**	**Sensitivity**	**Specificity**
Stage-1	Inferior lingular segment of L1	0.683	0.562	0.786
	Lateral segment of R2	0.721^*^	0.937	0.5
	Medial segment of R2	0.662	0.781	0.524
Stage-2	Apical segment of R1	0.662	0.778	0.625
	Posterior segment of R1	0.729	0.815	0.687
	Anterior segment of R1	0.755^*^	0.556	0.906
	Superior segment of R3	0.689	0.556	0.781
	Anterior basal segment of R3	0.72	0.704	0.719
	Medial basal segment of R3	0.69	0.519	0.875
	Lateral basal segment of R3	0.699	0.741	0.594
	Posterior basal segment of R3	0.659	0.704	0.625
	Superior lingular segment of L1	0.634	0.407	0.875
Stage-4	Anterior segment of R1	0.705	0.769	0.639
	Apical segment of R1	0.669	0.462	0.861
	Posterior segment of R1	0.734^*^	0.577	0.889
	Superior segment of R3	0.674	0.538	0.833
	Medial Basal segment of R3	0.678	0.885	0.417
	Apicoposterior segment of L1	0.707	0.692	0.778
	Anterior segment of L1	0.657	0.5	0.833

* *Represents the lung segment that contributes the most to lung involvement at each stage. R1, right upper lobe; R2, right middle lobe; R3, right lower lobe; L1, left upper lobe; L2, left lower lobe*.

**Figure 5:**
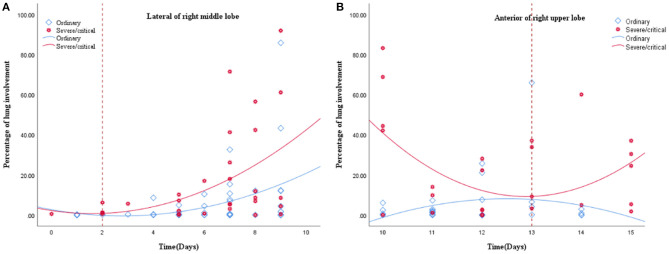
**(A,B)** show the fitted line of the lung involvement of the lateral segment of the right middle lobe and anterior segment of the right superior lobe in the first and second stages, respectively. The fit line formula of the first stage is Y = 2.23 – 1.88 * x + 0.59 * x2 (R2 = 0.213, *P* = 0.031), and the formula of the second stage is Y = 6.42E2–98.18 * x + 3.81 * x2 (R2 = 0.238, *P* = 0.038), in which x = days from the onset of the initial symptoms, y = percentages of lung involvement of lateral segment of right middle lobe and anterior segment of right upper lobe, respectively. The fit line plot shows that lung involvement accelerated on days 2 and 13 for the ordinary group and severe/critical group, respectively.

**Figure 6:**
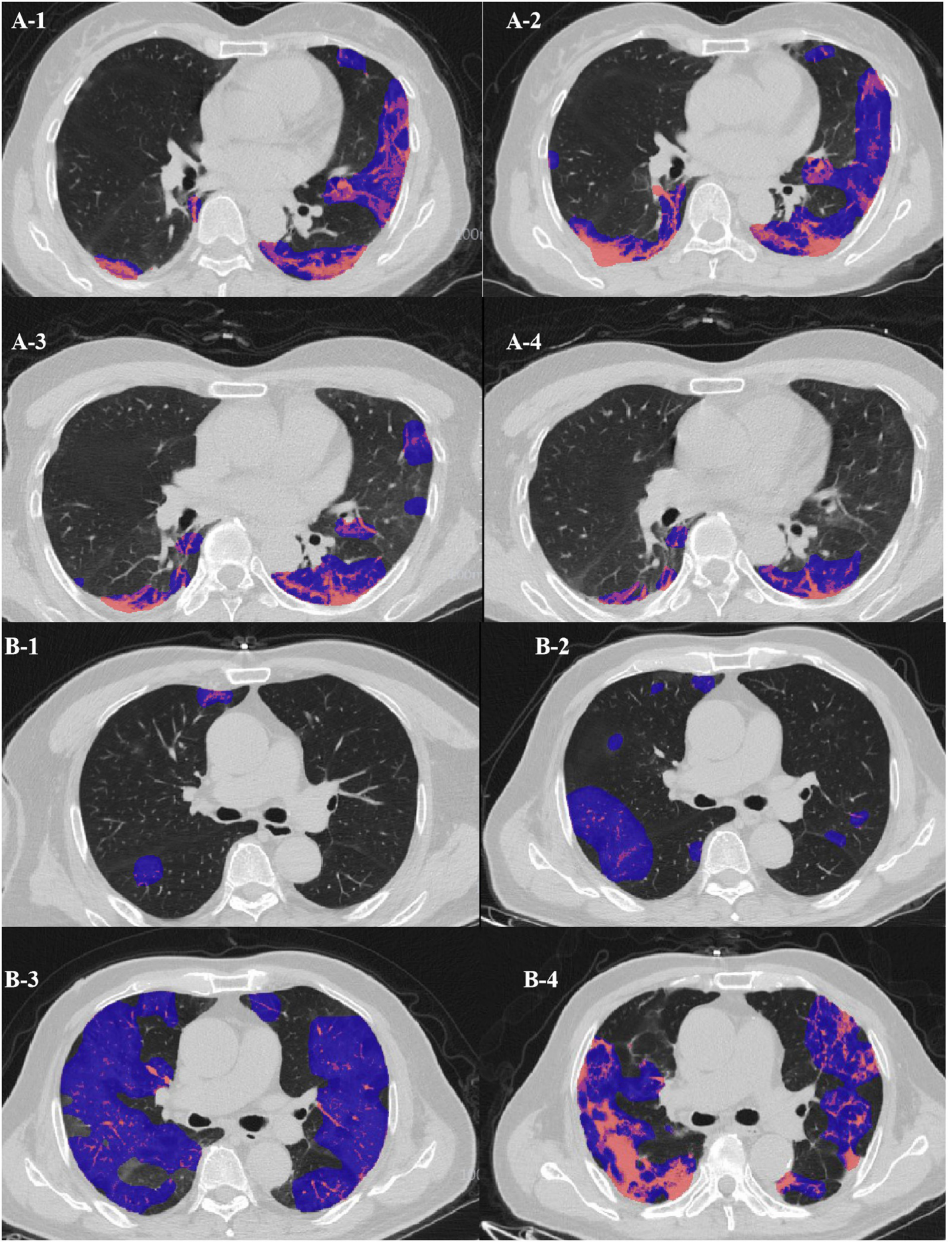
**(A)** Evolution of CT findings in a 51-year-old female patient of the clinically ordinary type presenting with persistent fever (37.8°C) for 5 days. **(A-1)** At the presentation (stage 1, day 5), with a PLI of 18.11%, showing a small region of subpleural GGO with partial consolidation mainly in the left upper lobe; **(A-2)** stage 2 day 10, with a PLI of 25.48%, a region of GGO enlarged while the density decreased; **(A-3)** stage 3 day 16, the density of the GGO further resolved, with a PLI of 14.71%; **(A-4)** stage 4 day 28, with a PLI of 8.93%, resolution with minimal residual GGO and parenchymal bands. **(B)** Evolution of CT findings in a 68-year-old male patient of the clinically severe type presenting with persistent fever (38.3°C) for 3 days, and with coronary heart disease for 5 years. **(B-1)** At presentation (stage 1, day 2), with a PLI of 2.7%, only small patchy GGO was visible; **(B-2)** stage 2 day 11, with a PLI of 12.7%, progressed into large patchy region of GGO; **(B-3)** stage 3 day 18, with a PLI of 57.75%, bilateral “white lung”; **(B-4)** stage 4 day 23, with a PLI of 48.87%, the “white lung” resolved obviously.

The scatter graph shows that the progress of the lateral segment of the right middle lobe was faster in the severe/critical group than that of the ordinary group from the second day in the first stage. In the second stage, the progress of the anterior segment of the right upper lobe was faster in the severe/critical group than that of the ordinary group on the 13th day, as shown in Figure 5. The fitted line of the fourth stage did not show statistical significance. Examples of the lung involvement evolution of two patients on CT images detected by AI software are shown in Figure 6.

## Discussion

Our study showed that age is the most important factor in differentiating the ordinary and severe/critical patients by demographics and clinical characteristics, which further supports previous research showing that older adults with COVID-19 are more likely to suffer from severe disease and have a poor prognosis (10). In addition, we found that the time to the lung involvement peak of the severe/critical patients is earlier than that of ordinary patients, which indicates that the severe/critical patients progress more swiftly, maybe due to the existence of a cytokine storm syndrome (11). Finally, we identified two major meaningful lung segments and timepoints in severe/critical patients. The lung involvement in the lateral segment of the right middle lobe on the second day and anterior segment in the right upper lobe on the 13th day after onset may suggest the possibility of potentially severe patients. Of note, the percentage of lung involvement had a high correlation with CT score, which suggested that the AI system we applied is highly reliable, which is consistent with other studies that the AI system can be a supplementary diagnostic method for clinicians (12).

This study showed that although age is an important indicator, it can only act as a risk factor during follow-up and was unable to predict short-term disease development. CT imaging can be used as a powerful predictive tool for early warning of short-term disease progression during follow-up. However, it needs to be emphasized that CT is a monitoring method for use only during a special period, and it is still necessary to consider the radiation damage of CT. Therefore, it is significant to identify the key timepoints of the disease for optimizing the frequency of CT examinations.

In this study, we first used the traditional visual CT score to analyse the entire treatment timeline and it showed that the peak time of lung involvement in the ordinary and severe/critical groups were 18 and 14 days, respectively. The time to the peak of severe/critical disease is obviously shorter, which may be related to the existence of a cytokine storm syndrome. The peak time of bilateral lower lung involvement of severe/critical patients was in the second stage (10–15 days), which is consistent with the results of the study by Heshui et al. (13), who found that the disease reached a peak within 2 weeks after onset and was mainly in the lower lobe. This may be because viral pneumonia mainly involves the lower lobes (14).

Furthermore, our study based on average PLIs at each stage further confirmed our results, that is, whether in the ordinary group or the severe group, the lung involvement of the bilateral lower lobes was greater than of the other lobes. In the severe group, we found the lung involvement peaks were during the second stage, and they were significantly higher than that of the ordinary patients at the same stage, which further shows that the severely ill patients had experienced rapid development in the second stage. However, lung involvement in the right middle lobe reached its highest in the fourth stage, and we speculate that this may be related to the lateral segment of the right middle lobe progressing rapidly in the first stage, staying stable during the later stage and then being absorbed slowly. A systematic review by Sana et al. (15) demonstrated that the greatest severity of CT findings was visible around day 10 after symptom onset, which is similar to the peak in our severe/critical group but a little earlier than that of our ordinary group. They showed that the imaging signs resolved after week 2 of the disease, which is also consistent with our results for the severe/critical group progressing earlier than the ordinary group.

In addition, we found a strange phenomenon. Among the five lobes, the peaks of the right upper, middle, and left upper lungs all occurred in the fourth stage, while the two lower lung involvement peaks occurred in the second stage. During the development of the disease, we speculate that the infection route of COVID-19 may develop from the bottom of the lung to the tip of the lung. This is significantly different from the lung involvement progression of ordinary patients, which occurs at the third stage peak. This may also be an early warning of the development of severe patients.

To further clarify the progress of severe patients in different precise anatomical locations in the lung lobes, it is necessary to evaluate the segmental lung involvement. Recent studies on COVID-19 have shown that the superior and posterior basal segments of the lower lobe of the lungs are the main locations affected by COVID-19 (16). However, due to the small range of lung segments, the use of traditional semi-quantitative and descriptive assessments of the evolution of the lesions in the corresponding small lung segments may have large deviations, especially for severe patients, and thus subjective evaluation may not reveal the dynamic relationship between development of the disease and time. Therefore, quantitative research using an AI system may be more accurate in revealing small-scale longitudinal lesion changes such as in the lung segments. Although AI cannot replace expert assessment at this stage, AI can provide considerable information to help clinicians perform rapid and complex decision-making. In this study, our results show a correlation between the percentage of lesions extracted by AI and the percentage of lesion involvement manually evaluated exceeds 0.8, which proves the reliability of the AI intelligent recognition system in identifying the lesions of COVID-19 pneumonia patients in this study. The AI-based quantitative analysis results show that the lesions in the anterior segment of the right upper lobe of the severe type in the second stage were significantly accelerated compared with the ordinary type.

Another result of this study is that the peak of the percentage of lung lesions in severe patients appeared in the second stage, and during the entire lesion change of right upper lobe over time, the peak of the second stage was already close to the peak of the fourth stage, so we speculate the lesions in the upper lobe of the right lung, especially the changes in the anterior segment lesions, may be an important sign of the accelerated progression of severe patients in the advanced stage. The mechanism that causes the rapid progression of the lesion may also be related to the anatomical structure of the upper lung lobe. An earlier study showed that when the lung is full of secretions, due to the anatomical position of the upper lung lobe, the vertical gradient of lung blood flow will affect the upper lobe ventilation or asymmetric perfusion, which leads to repeated infections and scars more likely to occur in the lower ventilated upper lobe (17). The pathological autopsies of patients who died from COVID-19 showed that there is a large amount of mucus secreted between the alveoli (18), so the progression of the lesions in the upper right lobe, especially the anterior segment, may further reflect this pathological mechanism. The peak of pneumonia in severe patients at 14 days also further supports this pathological mechanism, which may indicate the short-term effect of an inflammatory storm.

In addition, we found that the lateral segment of the right middle lobe in the first stage is also a typical anatomical site for severe pneumonia. Studies have shown that bronchiectasis caused by atypical infection is more serious in the right middle lobe, usually manifesting as bronchiectasis and atelectasis. It has also been shown that bronchiectasis has a greater impact on the middle lobe of the right lung (19, 20). Existing COVID-19 research has shown that traction bronchiectasis may reflect the viral load and virulence of COVID-19, and it is more common in emergency patients (21). The time curve shows that the lesions of the right middle lobe of the right lung in the first stage of severe patients progressed significantly faster than in ordinary patients, so we speculate that bronchiectasis in the early stage, especially on the second day, may be an early warning message for potentially severe patients.

Another study performed at the lung segment level also showed that COVID had a predominance of bilateral lower distribution, which is consistent with the previous descriptive studies (9, 21) and may be due to the physiologic characteristics of the lung caused by regional inhomogeneity as a result of the influence of gravity (22). However, the anterior segment in severe cases also progresses rapidly, and this may be attributed to many severe patients needing to lie down due to insufficiency of effective oxygen from their severe pneumonia, which then aggravates the lung involvement. This study further explored lung segment involvement and identified the most suggestive lung segments and their corresponding time for potentially severe/critical patients. Another explanation for this finding could be the fact that viral infections are prone to involve the lower lungs (13). A previous study showed CT signs of aggravation and repair coexisted in advanced-phase disease. Our graph of this study showed the lung involvement in all lobes in the ordinary group in the last stage resolved more obviously than those in the severe/critical group, which may be due to the weaker organ function in the elderly patients in the severe/critical group (23).

Our study has some limitations. First, the sample size is small, and pediatric patients are not included. Therefore, the results may only represent a specific age group of COVID-19 patients. Second, we do not have any treatment information about these patients, which may affect the patients' time course and CT quantification results. Finally, severe and critical cases were studied as one whole group. We may consider an independent study of critical cases in the future.

In conclusion, lung involvement in the lateral segment of the right middle lobe in the early stage or the rapid progression of the anterior segment of the right superior lobe in the middle stage is highly suspicious for a poor conversion, which can be used as a warning message for severe/critical patients.

## Data Availability Statement

The original contributions presented in the study are included in the article/Supplementary Material, further inquiries can be directed to the corresponding author/s.

## Ethics Statement

The studies involving human participants were reviewed and approved by Ethics committee of Wuhan Wuchang Hospital. Written informed consent for participation was not required for this study in accordance with the national legislation and the institutional requirements. Written informed consent was waived for this was a retrospective study, which involved no potential risk to the patients.

## Author Contributions

XY, GX, and SZ designed the study. YL, SZ, and XY performed the data acquisition and analysis. XY and SZ drafted and wrote the manuscript. All authors contributed to the article and approved the submitted version.

## Conflict of Interest

The authors declare that the research was conducted in the absence of any commercial or financial relationships that could be construed as a potential conflict of interest.

## Footnotes

^1^https://covid19.who.int/.
