# Supplementary material for: Early Warning Information for Severe and Critical Patients With COVID-19 Based on Quantitative CT Analysis of Lung Segments
Source: Front Public Health. 2021 May 13;9:596938. doi: 10.3389/fpubh.2021.596938 (PMC8155286; doi:10.3389/fpubh.2021.596938)
Supplement: Supplementary file 1 [file Table_1.DOCX]

1. **Diagnostic criteria of COVID-19 pneumonia**

## Case Definition for Surveillance of Coronavirus Disease 2019 (COVID-19) by Chinese Health Commission

| Suspected case  Present at least two of the following conditions of   1. Fever and/or respiratory symptoms (e.g., cough, myalgia, fatigue) 2. Imaging features of viral pneumonia 3. Normal or low white blood cell count or reduced lymphocyte in early-onset   AND  One or more of the following exposures during the 14 days before the onset of symptoms 1). Travel or residence history in Wuhan, other areas with recent local transmission of  COVID-19, or the local community with a confirmed patient   1. Close contact* with a patient with laboratory-confirmed COVID-19 (positive for the nucleic acid test) 2. Close contact with people from Wuhan or surrounding areas or local communities with fever or respiratory symptoms case report 3. Cluster onset   Patients without exposure history should meet all of the conditions i, ii and iii. |
| --- |
| Clinically diagnosed case (added in the trial fifth edition but deleted in the trial sixth edition)  The suspected case with typical imaging findings of pneumonia (only for Hubei) |
| **Confirmed case**  Suspected cases have at least one of the following etiological evidence   1. Positive real-time fluorescence polymerase chain reaction of the patient’s respiratory or blood specimen for COVID-19 nucleic acid 2. Viral gene sequences in respiratory or blood specimen are highly homologous to   COVID-19 |

*Close contact is defined as healthcare-related exposures, including direct care for patients with confirmed COVID-19, collaboration with healthcare workers with confirmed COVID-19, visiting or staying in the same closed environment with patients with confirmed COVID-19, or members who live in the same family environment with patients with confirmed COVID-19.

**2. Clinical classifications of COVID-19 disease**

The diagnostic criteria for COVID-19 and the determination of ordinary, severe and critical type are based on the seventh edition of COVID-19 issued by the National Health Commission of the People’s Republic of China,

The diagnostic criteria of COVID-19 disease were clinical manifestations and real-time fluorescence polymerase chain reaction revealed positive detection of COVID-19 in throat swabs or lower respiratory tract. Ordinary type is defined as having febrile and/or respiratory symptoms and imaging findings of pneumonia. Severe type is defined as patients suffer from shortness of breath, respiratory frequency ≥ 30/minute, or blood oxygen saturation ≤93%, or PaO2/FiO2 ratio <300. Critical type is defined as patients who suffered respiratory failure, septic shock, and/or multiple organ dysfunction/failure.

**3. Lung Segments**

Five lobes of the whole lung are divided into 18 segments，It includes the apical, posterior and anterior segments of the right upper lobe. The medial and lateral segments of the right middle lobe. The inner basal segment, anterior basal segment, outer basal segment, posterior basal segment and dorsal segment of the right lower lobe. The apical posterior segment, anterior segment, upper lingual segment and lower lingual segment of the left upper lobe, the dorsal segment, anterior internal base segment, outer base segment and posterior segment of the left lower lobe.
